# Exploring the automaticity of language-perception interactions: Effects of attention and awareness

**DOI:** 10.1038/srep17725

**Published:** 2015-12-07

**Authors:** Jolien C. Francken, Erik L. Meijs, Peter Hagoort, Simon van Gaal, Floris P. de Lange

**Affiliations:** 1 Radboud University, Donders Institute for Brain, Cognition and Behavior, Nijmegen, Netherlands; 2 Max Planck Institute for Psycholinguistics, Nijmegen, Netherlands; 3 Department of Psychology, University of Amsterdam, Netherlands

## Abstract

Previous studies have shown that language can modulate visual perception, by biasing and/or enhancing perceptual performance. However, it is still debated where in the brain visual and linguistic information are integrated, and whether the effects of language on perception are automatic and persist even in the absence of awareness of the linguistic material. Here, we aimed to explore the automaticity of language-perception interactions and the neural loci of these interactions in an fMRI study. Participants engaged in a visual motion discrimination task (upward or downward moving dots). Before each trial, a word prime was briefly presented that implied upward or downward motion (e.g., “rise”, “fall”). These word primes strongly influenced behavior: congruent motion words sped up reaction times and improved performance relative to incongruent motion words. Neural congruency effects were only observed in the left middle temporal gyrus, showing higher activity for congruent compared to incongruent conditions. This suggests that higher-level conceptual areas rather than sensory areas are the locus of language-perception interactions. When motion words were rendered unaware by means of masking, they still affected visual motion perception, suggesting that language-perception interactions may rely on automatic feed-forward integration of perceptual and semantic material in language areas of the brain.

Visual perception arises by an interaction between bottom-up sensory information and several top-down factors, such as attention and expectations^1^. Language has been suggested to be one such important top-down factor that can directly influence perception^2^,^3^,^4^. However, it is still debated where in the brain visual and linguistic information are integrated. One possibility is that linguistic information is processed in language-specific regions and then feeds back to lower-level sensory regions to modulate perceptual information processing^2^,^5^,^6^. Alternatively, language might influence perception at a later conceptual or decision stage rather than at the sensory stage^7^,^8^.

In a previous study, we investigated the effects of motion language on visual motion detection in a combined behavioral and fMRI study^8^. Participants were faster and more accurate when the direction implied by a motion word was congruent with the direction of a visual motion stimulus. We observed a neural counterpart of this behavioral facilitation effect in the left middle temporal gyrus (lMTG), an area involved in lexical retrieval, including word semantics and multisensory processing and integration^9^. These results are in line with an interaction of language and perception at a conceptual (semantic) processing stage.

In the current study, we aimed to further explore the automaticity of language-perception interactions and the neural loci of this interaction. First, we were interested in the effects of language on visual perception when motion words are attentively processed. In our previous study, motion word primes were irrelevant and ignored. It is conceivable that attentive processing of linguistic material may be necessary for more robust and widespread language-perceptual interactions^10^. Second, we tested whether language-perception interactions are dependent on awareness of the linguistic stimuli, i.e., if language still affects perception when participants are unaware of the motion words, in terms of brain and behavior.

To this end, we asked participants to discriminate moving dot patterns (upward or downward moving dots), which were preceded by congruent or incongruent motion word primes (e.g., “rise”, “fall”) (see Fig. 1a). To ensure attention on the linguistic prime stimuli, we added a concurrent semantic categorization task, to maximize processing of language stimuli and to enable the possibility for language-perception interactions (on 10% of the trials). To study the neural effects of awareness on language-perception interactions, we manipulated the awareness level of the motion words by backward masking. We previously found that linguistic primes still affect perception when they were perceptually invisible^11^, suggesting that language-perception interactions can occur independent of awareness. In the current fMRI study, we similarly manipulated awareness in order to directly study the neural locus of the congruency effect for aware and unaware language primes.

Finally, we were interested in the potential lateralization of language-perception interactions. Previous studies have indicated that these interactions might be larger, or exclusively present, for visual information processed in the language-dominant left hemisphere^8^,^12^, although evidence is mixed^13^,^14^,^15^. Therefore, we explored whether language primes had a stronger effect when visual stimuli were presented in the right hemifield (i.e., when processed by the left hemisphere).

## Results

### Behavioral results

In the semantic categorization (catch) task, discrimination performance of the masked words was at chance-level (binominal test, *p* > 0.05) for all participants. The discriminability of the words (motion vs. no motion) was markedly lower when the words were masked (unaware condition) than when they were not masked (aware condition) (difference *t*(22) = 9.31, *p* < 0.001; unaware *d’* = 0.00, corresponding to 49.7% correct responses, *t*(22) = 0.02, *p* = 0.99; aware *d’* = 2.20, 85.1% correct, *t*(22) = 8.75, *p* < 0.001). Thus, the masking procedure yielded clearly distinct aware and unaware conditions.

**Figure 1:**
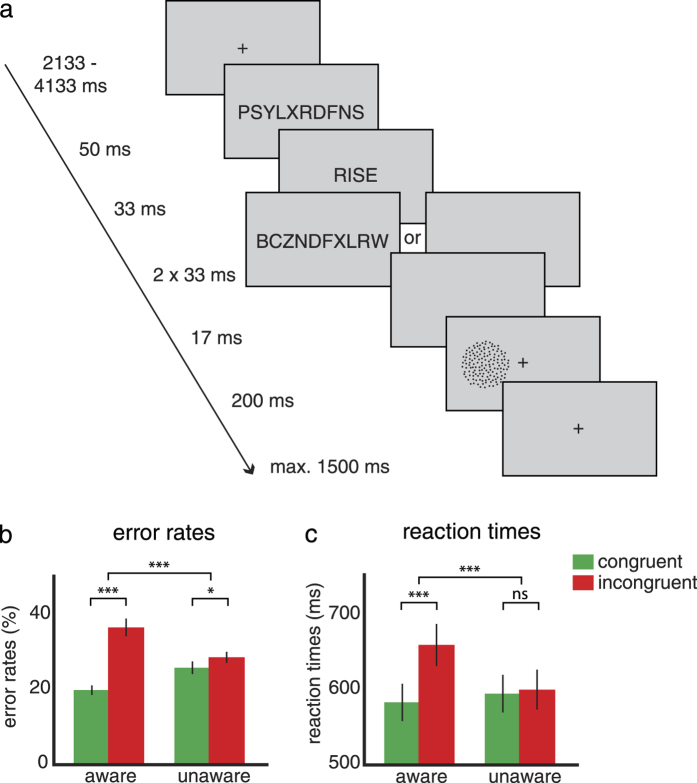
Experimental design and behavioral results. (**a**) A congruent or incongruent motion word (upward or downward, e.g., “rise“, or “fall“) is displayed in advance of every motion discrimination trial. All words are preceded by a forward mask; unaware words are additionally followed by two backward masks. The visual motion stimulus is presented either in the left or right visual field and the dots move upward or downward. In 10% of the trials the motion discrimination task was followed by an additional semantic categorization task (motion or non-motion) on the words. (**b**) Mean error rates (%) in the unmasked (aware, left bars) and masked (unaware, right bars) conditions for congruent (green) word-motion pairs were faster than incongruent (red) word-motion pairs. (**c**) Mean reaction times (in ms) in the aware condition, but not the unaware condition, were lower for congruent than incongruent motion words. n = 23, error bars denote SEM. *p < 0.05, **p < 0.01, ***p < 0.001, ns: not significant.

In the motion discrimination task, participants answered 72.1% of trials correctly (±5.8%, mean ± SD) at an average motion coherence level of 48.7% (17.7%, mean ± SD). Participants made fewer errors in discriminating the motion stimuli when they were preceded by a congruent motion word than by an incongruent motion word (main effect of congruency: *F*_1,22_ = 31.32, *p* < 0.001). This congruency effect was modulated by word awareness (congruency x awareness: *F*_1,22_ = 25.52, *p* < 0.001), indicating that the difference between congruent and incongruent conditions was larger when the words were unmasked than when they were masked. The congruency effect was clearly present when the words were unmasked (congruent: ER = 20.0%; incongruent: ER = 36.8%; ΔER = 16.8%, *F*_1,22_ = 32.18, *p* < 0.001; see Fig. 1b), but crucially, it was also present when the words were masked (congruent: ER = 26.0%; incongruent: ER = 28.8%; ΔER = 2.8%, *F*_1,22_ = 5.68, *p* = 0.026).

Congruency effects in reaction times showed a similar pattern. Participants responded faster to congruent compared to incongruent trials (main effect of congruency: *F*_1,22_ = 53.53, *p* < 0.001). The congruency effect was larger for unmasked words than for masked words (congruency x awareness: *F*_1,22_ = 57.91, *p* < 0.001) and was only significant for the unmasked trials (aware: congruent: 584 ms; incongruent: 661 ms; ΔRT = 77 ms, *F*_1,22_ = 82.22, *p* < 0.001; unaware: congruent: 595 ms; incongruent: 601 ms; ΔRT = 6 ms, *F*_1,22_ = 0.66, *p* = 0.43; see Fig. 1c).

In addition, we tested whether the congruency effect for masked and unmasked conditions was differentially modulated by the visual field in which the visual motion stimuli were presented. For both RTs and ERs, there was no interaction between congruency, awareness and visual field (both ps > 0.3; ERs: aware: ΔER LVF: 18.4%, ΔER RVF: 16.4%; unaware: ΔER LVF: 2.9%, ΔER RVF: 1.9%; RTs: aware: ΔRT LVF: 73 ms, ΔRT RVF: 85 ms; unaware: ΔRT LVF: 12 ms, ΔRT RVF: −1 ms).

In sum, both masked and unmasked words affected visual motion discrimination, although the effects were stronger for unmasked words.

### fMRI results

**Figure 2:**
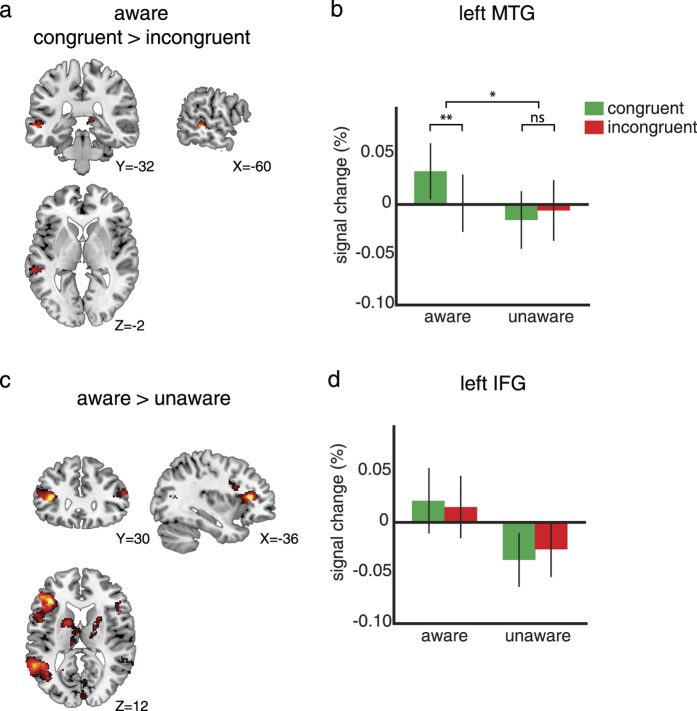
fMRI results. (**a**) The contrast between congruent and incongruent conditions plotted on frontal, sagittal, and transversal slices of an MNI brain (p < 0.01 uncorrected for illustration purposes). The only significant modulation because of congruency is localized in lMTG (n = 23). (**b**) Within the lMTG ROI (based on the independent language localizer) the percentage signal change for the congruent (green) and incongruent (red) conditions is plotted for both the aware (left) and unaware (right) conditions. Only the aware condition shows a congruency effect. (**c**) The contrast between aware and unaware conditions shows significantly more activation in the lMTG and lIFG for the aware condition (p < 0.01 uncorrected for illustration purposes). (**d**) Within the lIFG region from the contrast between aware and unaware conditions, the percentage signal change for the congruent and incongruent conditions is plotted. There is no modulation of congruency for either the aware or the unaware condition.

We next examined the neural locus of the behaviorally observed interaction between language and perception. We a priori identified two ROIs in the visual areas (l/r hMT+/V5) and one in the “language” areas (lMTG) as possible loci of this interaction (see Methods). Only in lMTG an effect of motion words on visual motion perception was observed (MNI coordinates peak voxel: [−60,−32,−2]). In this region, we observed a significant increase in activation for the congruent compared to the incongruent condition for the unmasked condition (See Fig. 2a,b, *F*_1,22_ = 8.80, p = 0.007). This congruency effect was significantly larger for the unmasked than for the masked conditions (congruency x awareness: *F*_1,22_ = 4.63, p = 0.043), and in the unaware condition no such effect was found (congruency: *F*_1,22_ = 0.53, p = 0.47). The size of the congruency effect for unmasked or masked conditions was not different for LVF compared to RVF stimuli (congruency x awareness x visual field: *F*_1,22_ = 1.46, p = 0.24) and there was no reliable positive correlation between congruency effects in behavior and neural congruency effects in the lMTG (RTs: r_s_ = 0.06, *p* = 0.77; ERs: r_s_ = 0.08, *p* = 0.72).

The left and right visual motion areas both showed a main effect of visual field (lhMT+/V5: *F*_1,22_ = 29.19, p < 0.001; rhMT+/V5: *F*_1,22_ = 7.55, p = 0.012; see Supplementary Figure S1 online), but in contrast to lMTG, no congruency effects were observed for either unmasked or masked conditions (lhMT+/V5: main effect of congruency: *F*_1,22_ = 0.07, p = 0.79; congruency x awareness: *F*_1,22_ = 0.51, p = 0.48; rhMT+/V5: main effect of congruency: *F*_1,22_ = 0.11, p = 0.75; congruency x awareness: *F*_1,22_ = 0.00, p = 1.00).

In addition, we were interested in potential differences between unmasked and masked conditions. This whole-brain contrast (corrected for multiple comparisons, see Methods) revealed stronger activation in two brain areas: the left inferior frontal gyrus (lIFG, MNI coordinates: [−36,30,12], *T*_22_ = 5.99, p < 0.001) and in the left middle temporal gyrus (lMTG, MNI coordinates: [−56,−40,8], *T*_22_ = 5.16, p < 0.001) that was previously identified by the language localizer (See Fig. 2c). A post-hoc ROI analysis revealed that the lIFG was not sensitive to the congruency between the motion word and the visual motion stimulus (main effect of congruency: *F*_1,22_ = 0.05, p = 0.83; congruency x awareness: *F*_1,22_ = 0.57, p = 0.46; see Fig. 2d). Finally, in a whole-brain analysis we confirmed that the congruency effect was specific to the lMTG, since no other regions showed a congruency effect (congruent > incongruent or incongruent > congruent) under either unmasked or masked conditions.

Together, the fMRI data show that only the lMTG is sensitive to the difference between congruent and incongruent motion words in a visual motion perception task, showing a stronger response when language primes and visual motion signals correspond. These effects were only observed when motion words were consciously perceived.

## Discussion

We investigated the dependence of language-perception interactions on awareness and the neural loci of this interaction. In a visual motion discrimination task in which attention was explicitly directed to motion word primes, congruent motion words significantly sped up reaction times and improved performance relative to incongruent motion words. Despite the large behavioral effects, language-perception congruence affected only the lMTG, where neural activation was higher for congruent compared to incongruent conditions. This neural congruency effect was obliterated by masking the words, even though a small behavioral effect persisted.

In a previous study, we investigated behavioral and neural effects of motion words on visual motion detection^8^. When comparing those results to our current findings, we notice that the behavioral congruency effects in the current study were much stronger: in the previous study, the difference between congruent and incongruent conditions was on average 20 ms (RTs) and 4% (ERs), while in the current study the difference was more than three times larger (70 ms and 15%). This difference is easily explained by the fact that the current study included an additional semantic categorization task on the motion words, while in the previous study the words were task-irrelevant and therefore unattended. We next examined whether increased attention to the motion words also resulted in an interaction of language primes and visual stimuli in a wider network of brain areas, feeding back to sensory areas.

Interestingly however, the only brain region that was sensitive to the difference between congruent and incongruent conditions was the lMTG, replicating our previous results^8^. Based on these results we conclude that motion sensitive sensory areas (i.e., hMT+/V5 and more ventral parts of the visual cortex) do not seem to be involved in the integration of linguistic and perceptual information, even when both are thoroughly processed, contrary to suggestions from previous studies. First, corresponding with top-down theories of visual perception, words could induce an expectation about the visual world, thereby automatically recruiting the relevant sensory areas^1^,^2^. According to one influential account, predictive coding, higher-order regions send predictions to lower-order regions, and these predictions are then compared to the sensory evidence. When they match, a small prediction error results and this induces a relative decrease in neural activation, whereas when there is a mismatch, a large prediction error leads to increased activation^16^,^17^,^18^,^19^. However, the hypothesis that language affects perception by producing an expectation was not supported by our data, possibly because we used non-predictive linguistic cues (i.e., an equal number of congruent and incongruent conditions). Second, effects in the visual cortex would also be expected according to studies investigating the neural effects of reading or listening to motion- or action-related language^10^,^20^,^21^,^22^,^23^,^24^,^25^,^26^. Our study differs from these previous studies in one critical aspect: we included both linguistic and visual material, since we were interested in the effects of motion language on perception. As a consequence, participants had to focus on a challenging motion discrimination task, instead of on the motion words, which might have resulted in more superficial semantic processing. Alternatively, a response priming explanation, suggested by studies exploring the role of visual information in action recognition and execution (e.g.^27^), is also unlikely since we do not find any evidence for involvement of decision- or response conflict-related brain areas. Our findings rather provide evidence for a feed-forward model of language-perception interactions^11^. Within this model, motion words and motion signals are each processed in separate areas, which do not interact. Both signals activate a (common) conceptual representation (embodied in the lMTG), however, and it is here at the conceptual level that linguistic information interacts with the visual motion stimuli^28^. This suggests that semantic categorization may be an integral part of perceptual decision making^8^,^29^,^30^.

We further asked whether language-perception interactions can also occur outside of subjects’ awareness, since effects of unconscious stimuli on cognitive processing and behavior have been reported before (see for review^31^,^32^). In line with a previous study^11^, we provide support for this notion by showing larger error rates for invisible motion words that were incongruent with upcoming visual stimuli, compared to congruent motion words. We did not observe robust differences in brain activity between congruent and incongruent stimuli when the motion words were unconscious.

When comparing neural activity for consciously perceived vs. unconscious motion words, there was larger activity in a left-lateralized language network comprising the lMTG and the lIFG. Within this network, lMTG is implicated in lexical retrieval, including word semantics and multisensory processing and integration^9^,^33^,^34^,^35^,^36^,^37^ whereas the lIFG is involved in unification operations that maintain, select, and integrate multiple sources of information over time^33^,^35^,^36^. Of these two areas, larger effects of congruent relative to incongruent conditions were only observed in the lMTG, in line with previous studies^33^. We speculate that this is due to the fact that the congruence effect happens at the level of the conceptual representation, rather than the level of selection and maintenance of semantic material.

Finally, we were interested in the potential different effects of language on the processing of visual stimuli presented in the left visual field compared to the right visual field, since there has been mixed support for a lateralization of language-perception interactions^4^,^12^,^13^,^14^,^15^. In fact, using a highly similar design, we previously observed stronger behavioral congruency effects for stimuli presented in the RVF^8^ but equal congruency effects in the current study and a previous behavioral study^11^. A critical difference between these studies is the extent to which attention was paid to the language primes. We speculate therefore that lateralization of language-perception interactions may depend on the extent to which attention is directed to the language stimuli. Unattended stimuli may ‘remain local’ and thereby only affect visual processing in the same hemisphere leading to unilateral effects, whereas attended stimuli might be ‘broadcasted’ to other neural processors^31^,^38^, resulting in larger and bilateral effects. Future studies are required to directly assess the potential effects of attention on language-perception interactions.

In conclusion, we have explored the neural locus and behavioral characteristics of language-perception interactions for attended motion words, under different conditions of awareness. Motion words had large behavioral effects on visual perception. A neural counterpart of this integration process was observed in the lMTG, suggesting that higher-level conceptual areas, rather than sensory areas, are the locus of language-perception interactions.

## Methods

### Participants

Twenty-six healthy, right-handed participants with normal or corrected-to-normal vision (21 female, age 22.7 ± 2.9 years) took part in two experimental sessions. All participants were native Dutch speakers and reported having no reading problems. The experimental protocol was approved and all participants gave written informed consent in accordance with the declaration of Helsinki and guidelines of the local ethics committee (CMO region Arnhem-Nijmegen, The Netherlands). Compensation was approx. 50 Euros or course credit.

### Stimuli

Stimuli were generated using the Psychophysics Toolbox^39^ within MATLAB (MathWorks, Natick, MA, US), and displayed on a rear-projection screen using an EIKI projector (60 Hz refresh rate, 1024 × 768 resolution) in the fMRI experiment. Stimuli were presented in white on a light-gray background. The visual random-dot motion (RDM) stimuli consisted of white dots (density = 2.5 dots/deg^2^; speed = 6.0 deg/sec) plotted within a circular aperture (radius 7.5 deg). On every trial, the RDM stimulus was presented on either the left or right side of the screen (8.5 deg horizontal eccentricity from fixation to centre of circular aperture) for 200 ms. In the first frame of the RDM stimulus, a random configuration of dots was presented within the annulus. Subsequently, on every frame a certain percentage of the dots was replotted consistently in one direction (upward or downward) on the next frame (see Procedure). Dots moving outside of the annulus and other remaining dots were replotted at a random location within the annulus.

Five verbs describing each direction of motion (in Dutch, here translated to English; upward: *grow, ascend, rise, climb, go up;* downward: *sink, descend, drop, dive, go down*), and ten non-motion verbs (*bet, mourn, exchange, glow, film, rest, cost, sweat, wish, relax*) were used in the experiment. Motions words and neutral words were matched for lexical frequency (taken from the CELEX database) and word length (5–8 letters)(both p > 0.2). Masks were randomly generated combinations of ten consonant strings. Both words and masks were presented at the center of the screen, using capital letters in a mono-spaced font.

### Procedure

Participants performed a motion discrimination task (upward vs. downward motion) on a visual RDM stimulus (see Fig. 1a). A central fixation cross (width 0.4 degrees) was presented throughout the trial, except when a word, mask or blank screen was presented. Each trial started with a centrally presented forward mask (50 ms) followed by a word (33 ms), which could either be a motion word or a non-motion (neutral) word. Motion words were not predictive for the motion direction of the visual motion stimulus, i.e., after the word “rise” an upward visual motion stimulus was presented in 50% of the cases, resulting in an equal number of congruent and incongruent conditions. Presentation of the words was pseudorandom. Awareness of the word was manipulated by presenting either backward masks (2 × 33 ms; unaware condition) or a blank screen (67 ms; aware condition) after word presentation. A short inter-stimulus interval (ISI) of 17 ms was always present after either of these screens. Next, a visual RDM stimulus was presented (200 ms) in either the left visual field (LVF) or in the right visual field (RVF). Participants had to indicate whether the RDM contained upward or downward motion, while maintaining fixation at the central cross. The brief presentation time of the RDM stimulus served to minimize the chance of eye movements to the stimulus, as saccade latencies are in the order of ~200 ms^40^. Participants were instructed to respond as quickly and accurately as possible by pressing a button with either the index or middle finger of the right hand (counterbalanced across participants). The inter-trial interval (ITI) was 2133–4133 ms.

In 10% of the trials the motion discrimination task was followed by an additional semantic categorization task (motion or non-motion) on the words. These catch trials were included for two reasons. First, they ensured attention to the words, which enhances processing of the primes in both unmasked and masked conditions^41^,^42^. Second, catch trials were used to estimate word awareness. Participants were explicitly told that there was a fifty percent chance of either motion or non-motion words in the catch trials. Note that non-motion words were solely included to test for the visibility of the words.

The experiment consisted of two sessions on separate days within one week. In the first session participants performed a training phase outside of the scanner to familiarize them with the task. Participants first practiced the motion discrimination task in three blocks with fixed coherence levels (80%, 55%, and 30% respectively). In the next three training blocks, participants practiced the discrimination task while the words were presented and the catch task was added. The coherence level of the motion stimuli was individually adapted to performance in the previous blocks. In the second session, participants received one block of training within the scanner (motion coherence level from previous session) and the coherence level after this training block was taken as the starting point for a Bayesian adaptive staircase procedure^43^. This was done to yield comparable task difficulty and performance for all participants. The threshold for discrimination was defined as the percentage of coherent motion for which the staircase procedure predicted 75% accuracy. During training (except for the final training blocks and the threshold estimation block), we provided participants with trial-by-trial feedback for both the motion task and the catch trial task by means of a green or red fixation cross for correct and incorrect responses, respectively.

The actual experiment consisted of ten blocks of 50 trials (500 trials in total). In the experiment, the coherence level was fixed within a block, but the Bayesian staircase procedure was still running over the course of the 50 trials to obtain a new threshold estimate. After each block, this estimate was used to update the coherence level to accommodate potential practice and fatigue effects over the course of the experiment. Summary feedback (percentage correct) was provided during the break after each block.

We also acquired two additional localizer tasks. In the motion localizer, we presented the same motion stimuli that we used in the main experiment (see Stimuli). The motion coherence level was fixed to 100% and the duration of a trial was 16 s. There were ten blocks of seven trials each, presented in pseudorandom order. Motion presentation occurred in two directions (upward, downward) and at three different locations of the screen (left, center, right). Each combination of motion location and motion direction was present in every block, and counterbalanced across the trials in that block. The last trial of a block was always a fixation trial in which only a fixation cross was presented. The subject’s task was to press a button when the fixation cross turned from white to dark grey (two or three times during a trial, at random intervals), to help them fixate at the center of the screen. The central motion stimulus had an aperture radius of 9 degrees and a central aperture of 1 degree in which the fixation cross was displayed.

In the language localizer, we presented the same word lists that we used in the main experiment (see Stimuli), plus additional words from the training set, resulting in 10 different words per category. Subjects were presented with eighteen blocks (14 for the first participant) of five trials. Each trial consisted of 300 ms presentations of 25 words alternating with 300 ms fixation (15 s per trial, central presentation). Within a trial, all words were from the same category (upward, downward, non-motion, random letter strings (6–8 consonants) and a fixation condition). The order of trials within a block was pseudorandom, with the exception of the fixation trial, which was always the last trial of a block. Participants were instructed to monitor occasional word repetitions (1-back task, occurring 1–4 times per trial) to make sure that they would attentively read the words. Words were presented in the center of the screen. For both localizer tasks, the inter-trial interval was 1 s.

Three participants were excluded from the analyses for reasons outlined below. Performance of one participant on the motion discrimination task was at chance level, one participant had excessive head movement during scanning (>5 mm) and one participant showed a deviant pattern of language lateralization (right-hemisphere dominance). All analyses were performed on the remaining 23 participants.

### Behavioral analysis

We calculated congruency effects for reaction times (RT) on correct trials and error rates (ER). On congruent trials, the motion described by the word matched the direction of visual motion, e.g. “rise” was followed by a stimulus with upward moving dots. On incongruent trials, the motion described by the word and the direction of visual motion did not match. Missed trials and trials with RTs that were >3 SD than the individual subject mean RT were excluded from the analyses (in total 2.4%). Each of two behavioral measures was subjected to a repeated measures analysis of variance, including factors *Congruency* (congruent, incongruent), *Awareness* (aware, unaware) and *Visual Field* (LVF, RVF).

To assess the awareness of the words, we calculated accuracy and *d’* in the catch trials. *d’* is an unbiased measure of the discriminability sensitivity of the observer^44^. We used the accuracy in binomial tests to determine for every participant whether performance was above chance (50% correct). *d’* for the unmasked and masked conditions were subsequently compared to each other using paired t-tests and then compared with zero using one-sample t-tests.

### fMRI acquisition

Images were acquired on a 3.0 Tesla Skyra MRI system (Siemens, Erlangen, Germany). *T*_2_*-weighted gradient-echo echo-planar images (repetition time: 2000 ms, echo time 30 ms, 29 ascending slices, distance factor 20%, voxel size, 2 × 2 × 2 mm, flip angle 80 degrees, field of view 59 mm) were acquired using a 32-channel head coil. We chose to focus on the visual, middle/superior temporal and middle/frontal areas and we did not include the parietal, inferior temporal and motor cortices, guided by the results from our previous study^8^ and our hypotheses. The reason that we could not include the whole brain was a trade-off between voxel size, TR, and the number of slices, since we planned to do MVPA analyses on our fMRI data, which benefits from smaller voxel size. However, because decoding of motion direction on the basis of the activity patterns obtained during the motion localizer data was unreliable, we did not perform any further MVPA analyses. A high-resolution anatomical image was collected using a *T*_1_-weighted magnetization prepared rapid gradient-echo sequence (repetition time: 2300 ms, echo time 3.03 ms, voxel size 1 × 1 × 1 mm).

### fMRI data analysis

Analysis was performed using SPM8 (http://www.fil.ion.ucl.ac.uk/spm, Wellcome Trust Centre for Neuroimaging, London, UK). The first four volumes of each run were discarded to allow for scanner equilibration. Preprocessing consisted of realignment through rigid-body registration to correct for head motion, slice timing correction to the onset of the first slice, coregistration of the functional and anatomical images, and normalization to a standard *T*_1_ template centered in MNI space by using linear and nonlinear parameters and resampling at an isotropic voxel size of 2 mm. Normalized images were smoothed with a Gaussian kernel with a full-width at half-maximum of 8 mm. A high-pass filter (cutoff, 128 s) was applied to remove low-frequency signals, such as scanner drift. The ensuing preprocessed fMRI time series were analyzed on a subject-by-subject basis using an event-related approach in the context of the general linear model.

Regressors for the first-level analysis were obtained by convolving the unit impulse time series for each condition with the canonical hemodynamic response function. We modeled the 24 different conditions of the experiment [motion type (2) × word type (3) × visual field (2) × awareness (2)] separately for each of the two sessions. We did not compare the effects of non-motion words with motion words because the former were less frequent and occurred more often in catch trials. Catch trials and resting periods were each modeled as a regressor of no interest, as were head motion parameters^45^. For the localizers, we used the same procedure. Both localizers used a block design. The motion localizer had seven conditions and block duration of 16 s. The language localizer had five conditions and block duration of 15 s.

### Statistical analysis

We used *a priori* functional information on the basis of the results from the localizers to constrain our search space^46^. In particular, we isolated the regions that were involved in semantic language processing (language localizer) and visual motion processing (motion localizer). These corresponded to the left middle temporal gyrus (lMTG, language localizer) and bilateral hMT+/V5 (motion localizer).

Specifically, we obtained the anatomical location of the lMTG by contrasting the three word conditions (up, down, neutral words) with the random consonant letter strings condition (MNI coordinates: [−60,−26,2], voxel threshold of *p* < 0.001 uncorrected at the whole brain level). Likewise we obtained the search volume of the right hMT+/V5 ROI by contrasting visual motion stimulation in the LVF > RVF (MNI coordinates: [46,−72,0]) and combining this with a right hMT+/V5 anatomical template (Anatomy Toolbox SPM8), and we used the same procedure to obtain the left hMT+/V5 ROI (with the reverse contrast; MNI coordinates: [−42,−86,8]). We computed the mean activity over the voxels in each ROI for the different conditions and performed a repeated measures analysis of variance, including factors *Congruency* (congruent, incongruent), *Awareness* (aware, unaware) and *Visual Field* (LVF, RVF). Additional whole-brain statistical inference was performed using a cluster-level statistical test to assess clusters of significant activation^47^. We used a corrected cluster threshold of *p* < 0.05, on the basis of an auxiliary voxel threshold of *p* < 0.001 at the whole-brain level.

## Additional Information

**How to cite this article**: Francken, J. C. *et al*. Exploring the automaticity of language-perception interactions: Effects of attention and awareness. *Sci. Rep.* **5**, 17725; doi: 10.1038/srep17725 (2015).

## Supplementary Material

Supplementary Information
