# Supplementary material for: Exploring the automaticity of language-perception interactions: Effects of attention and awareness
Source: Sci Rep. 2015 Dec 7;5:17725. doi: 10.1038/srep17725 (PMC4671057; doi:10.1038/srep17725)
Supplement: Supplementary Information [file srep17725-s1.pdf]

**Supplementary Information**

# Exploring the automaticity of language-perception interactions: Effects of attention and awareness

Jolien C. Francken, Erik L. Meijs, Peter Hagoort, Simon van Gaal, Floris P. de Lange,

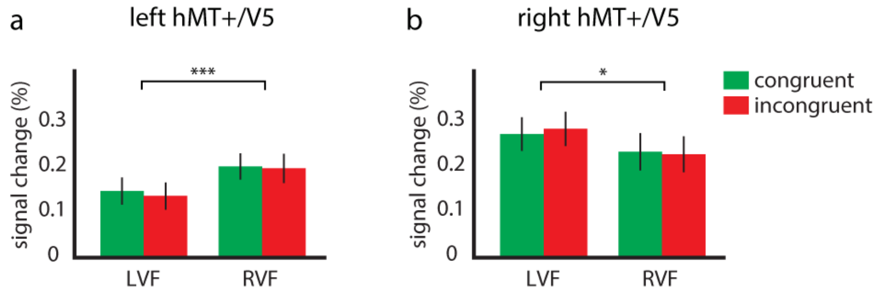


**Supplementary Figure S1. fMRI results**. (a) Within the lhMT+/V5 ROI the percentage signal change for the congruent (green) and incongruent (red) aware conditions is plotted for both the LVF (left) and RVF (right). (b) Congruency effects in the rhMT+/V5 ROI. Conventions as in (a). n = 23, error bars denote SEM. * p<0.05, *** p<0.001.
